# Joint models for dynamic prediction in localised prostate cancer: a literature review

**DOI:** 10.1186/s12874-022-01709-3

**Published:** 2022-09-19

**Authors:** Harry Parr, Emma Hall, Nuria Porta

**Affiliations:** Clinical Trials and Statistics Unit at The Institute of Cancer Research, London, UK

**Keywords:** Dynamic prediction models, Prostate cancer, PSA, Joint modelling, Dynamic predictions, Personalised medicine

## Abstract

**Background:**

Prostate cancer is a very prevalent disease in men. Patients are monitored regularly during and after treatment with repeated assessment of prostate-specific antigen (PSA) levels. Prognosis of localised prostate cancer is generally good after treatment, and the risk of having a recurrence is usually estimated based on factors measured at diagnosis. Incorporating PSA measurements over time in a dynamic prediction joint model enables updates of patients’ risk as new information becomes available. We review joint model strategies that have been applied to model time-dependent PSA trajectories to predict time-to-event outcomes in localised prostate cancer.

**Methods:**

We identify articles that developed joint models for prediction of localised prostate cancer recurrence over the last two decades. We report, compare, and summarise the methodological approaches and applications that use joint modelling accounting for two processes: the longitudinal model (PSA), and the time-to-event process (clinical failure). The methods explored differ in how they specify the association between these two processes.

**Results:**

Twelve relevant articles were identified. A range of methodological frameworks were found, and we describe in detail shared-parameter joint models (9 of 12, 75%) and joint latent class models (3 of 12, 25%). Within each framework, these articles presented model development, estimation of dynamic predictions and model validations.

**Conclusions:**

Each framework has its unique principles with corresponding advantages and differing interpretations. Regardless of the framework used, dynamic prediction models enable real-time prediction of individual patient prognosis. They utilise all available longitudinal information, in addition to baseline prognostic risk factors, and are superior to traditional baseline-only prediction models.

## Background

Prostate cancer is highly prevalent, as the 2nd most diagnosed cancer in men worldwide (1.3 million cases in 2018) [1] and the most common cancer among men in the UK [2, 3]. Within the UK, one in eight men on average are diagnosed with prostate cancer, increasing to 1-in-6 for men born after 1960. In the period 2014–16 there were almost 48,000 diagnosed per-annum and over 11,500 deaths in 2016 from prostate cancer [4–6]. Over half of patients (56.5—61.3%) present at diagnosis with localised prostate cancer, where disease is confined to the prostate and has not yet spread to the nodes or other organs of the body [7, 8]. Many treatment options are available to patients with localised prostate cancer, including external-beam radiotherapy (EBRT), brachytherapy, radical prostatectomy; or conservative management strategies for favourable-risk prostate cancer to delay or avoid aggressive treatment and potential side effects [9]. Potential treatment-related toxicities and side effects often affect management treatment choices [10]. EBRT is deemed most appropriate for those with moderate- or high risk disease [11]. A combination with hormonal therapy (HT) can be given for its neoadjuvant efficacy [9]: for low-grade localised prostate cancer, HT can be given 3–6 months before EBRT treatment, or longer for higher-risk stages [12].

Patients are monitored regularly during, and after treatment. In particular, for their prostate-specific antigen (PSA), a serine protease protein biomarker secreted by the prostate [13]. Repeated PSA readings are taken during patient check-ups. Patients present at diagnosis with elevated PSA levels, which decrease upon starting of HT and radiotherapy (RT). Thus, increased levels of PSA after treatment suggest a growth of prostate cancer cells, reflecting a higher risk of prostate cancer recurrence. PSA is used to determine biochemical failure (BcF), defined as a PSA concentration greater than the nadir (the lowest observed PSA value) plus 2 ng/mL [14]. HT can be used as a salvage therapy following BcF, lowering PSA levels and decreasing risk of clinical failure. Local or distant recurrence is confirmed by imaging.

There are known patient and tumour risk factors that affect prognosis of localised prostate cancer. These include PSA levels at diagnosis, tumour stage (as per the TNM scoring system) and Gleason score/grade grouping [15]. These risk factors are used to categorise patients into the National Comprehensive Cancer Network (NCCN) low, intermediate and high risk groups [16] (Table 1). Prognosis of localised prostate cancer (T1–T2N0M0) is generally good after treatment, with 5-year disease-free survival rates around 76% (95% CI: 75%—76%) [17].

**Table 1:** Risk stratification by clinical risk factors: Clinical T-stage, Gleason score, and presenting PSA. Locally advance prostate cancer includes high-risk localised patients, as defined by NCCN [16]

Risk level	Clinical T-stage	Gleason Grading Group	Presenting PSA	*Condition to be met*
Low risk	T1-T2a	1	< 10 ng/mL	*All three*
Intermediate risk	T2b-c	2 or 3	10-20 ng/mL	*Any*
High risk	T3a	4 or 5	> 20 ng/mL	*Any*

Clinical prediction models (CPMs) are developed from patient and tumour features at diagnosis, as well as information on short-term treatments, to predict future prognosis. To date, a plethora of CPMs guide management decisions for localised prostate cancer, visualised in nomograms and online calculators [18–26]. These CPMs only consider information available at the time of diagnosis and/or at start/end of treatment, and PSA values collected after that timepoint are rarely considered, if only for the definition of BcF. However, it is of interest to both patient and clinician to examine the association of the biomarker of interest over time to prognosis. Knowing the patient is alive and recurrence-free at the new visit, with an updated PSA value, is informative. Including this new information into a prediction model can elicit dynamic predictions that enable updated prognosis of patients.

A naïve approach would be to consider PSA as a time-dependent variable in an extended Cox/relative risk model [27]. However, this is not appropriate due to the endogenous nature of the biomarker of interest [28, 29], which contains biological variation and measurement error. A further extension is to use landmark modelling [28, 30–34]: dynamic predictions are obtained by fitting time-dependent Cox models to the patient subsample still at risk at several prediction, or landmark times of interest, together with the value of the longitudinal biomarker at that time. Landmark models are straightforward to fit with standard software, but no measurement error for the time-varying biomarker is considered nor is the entire longitudinal history of the biomarker utilised (due to using the last observation carried forward) [34]. To improve predictions, a two-stage approach to landmarking (also known as mixed model landmarking [28, 35]) can be considered to model measurement error and incorporate the full biomarker history. However, uncertainties in the mixed-effect model estimates are not carried through to the survival submodel, resulting in overexact estimates [36].

Joint models (JMs) permit dynamic prediction in localised prostate cancer by considering two time-dependent processes simultaneously: the repeated longitudinal PSA biomarker over time (modelled using a mixed-effects submodel), and the time to an event of interest (modelled using a relative-risk, or Cox submodel). The event of interest can be BcF, recurrence of disease (either locally in the prostate gland, in the regional lymph nodes or in a distant organ), clinical failure (need to re-commence HT), death, or a composite of all these events. The association between these two processes can be captured by shared random effects in both the longitudinal and time-to-event submodels (shared-parameter joint models, SPJMs) or by assuming a latent association structure between them (joint latent class models, JLCMs).

In this paper, we synthesize a review for published applications of joint models to localised prostate cancer over the last two decades, focusing on the modelling of the time-to-event process(es), the functional form of PSA, validation strategies and evaluation of dynamic predictions. We describe the search strategy to identify papers, and we briefly describe the joint modelling methodology, as well as how to compute dynamic predictions, measures of predictive performance from joint models. Given the rapid popularity and use of dynamic prediction models, this article serves as a reference to assess and reflect the applied and dynamic methods used in localised prostate cancer. The main review of the identified articles is given in the results section and summarised on Table 2. Finally, an appraisal and conclusion of these models are given.

**Table 2:** Summary table of joint modelling articles applied to localised prostate cancer and clinical failure, in chronological order

Paper [ref]	Modelling Framework	Sample sizes (N) & Events (E) for model development	Joint model parametrisation	Dynamic prediction landmark and prediction window	Validation undertaken	Code & software used
1) Pauler & Finkelstein, 2002 [37]	Bayesian change-point SPJM.	*N* = 676, E = 176	PSA data during the first two years was dropped from analysis due to rapid drops of PSA post-EBRT & HT. The random effects include the intercept and the slopes (before & after the change-point). The change-point indicator predicts recurrence. Logged-PSA is modelled with covariates age, presenting PSA, T-stage, with change-point indicator.	Change-point occurring within 10 years. Relapse landmark by four years with a prediction horizon of 10 years.	None performed.	C routine *dfpmin*, and S-PLUS *surv.fit* function.
2) Law et al., 2002 [38]	Frequentist cure SPJM.	*N* = 458, E = 92	Two models are fitted, joint-cure and logistic-Cox (no longitudinal PSA consideration). Nonlinear exponential- decay & growth modelled longitudinal logged-PSAs using presenting PSA, T-stage, and Gleason.	Not specified, estimated probabilities of recurrence are given for each patient at some time in the future.	Simulation study performed showing that joint-cure model has better sensitivity and discrimination compared to logistic-Cox model.	MATLAB
3) Yu et al., 2004 [39]	Cure SPJM (comparing Bayesian and Frequentist).	N = 458, E = 92	Modelled current PSA value and the PSA gradient trajectory. Random effects are modelled parametrically by exponential- decay & growth models adjusting for presenting PSA, T-stage, and Gleason.	Not specified, estimated probabilities of recurrence are given for each patient at some time in the future.	Not done – comparisons are made between the two estimation methods and are shown to be similar to one another.	MATLAB & C++
4) Taylor et al. 2005 [40]	Bayesian cure SPJM.	*N* = 934, E = 140	PSA value & slope and time-dependent hormone therapy commencement indicator is considered, adjusting for baseline covariates: presenting PSA, T-stage, Gleason, age, total dose (Gy), and treatment duration.	Landmarks from last contact, with a prediction window of four years.	Validation performed on data of the same patients used for development, but with further follow-up. The model is shown to be well calibrated and accurately predict new PSA values and recurrence risk.	C++
5) Yu et al., 2008 [41]	Bayesian cure SPJM.	*N* = 928, E = 146	PSA value & slope and time-dependent hormone therapy commencement indicator is considered, adjusting for baseline covariates: presenting PSA, T-stage, Gleason, age, total dose (Gy), and treatment duration.	Landmarks from last contact, with a prediction window of four years.	Validation performed on data of the same patients used for development, but with further follow-up. The model is shown to be well calibrated and accurately predict new PSA values and recurrence risk. Kaplan-Meier plot shows the higher predicted risks go on to have more recurrences indicating its validity.	C++
6) Proust-Lima & Taylor, 2009 [42]	Frequentist JLCM.	*Model development and validation:* *N* = 2386, E = 317	Baseline covariates included: presenting PSA, T-stage, and Gleason. The main- and random effects are of the biphasic initial decline and long-term rise. Five latent classes were identified.	Landmarks taken at every six months from 1—3½ years, with a prediction window of three years.	External validation of prediction is performed on two external cohorts. A range of models are explored, the 5-JLCM shows consistently lower absolute- and weighted prediction errors in both cohorts, using prediction windows of 1 and three years.	Not stated but presumably R using the *lcmm* package.
7) Jacqmin-Gadda et al., 2010 [43]	Frequentist JLCM.	*N* = 459, E = 74	Similar to [42] with biphasic longitudinal components for the logged-PSA, considering presenting PSA, T-stage, and Gleason. Four latent classes were identified to be best fitting where the proposed score test did not reject the null of conditional independence.	Only mean evolutions for each of the four classes are given with predicted recurrence-free survival. No windows are specified.	Simulation study performed to appraise score test, where baseline hazard function was misspecified. This methodology was applied to prostate cancer cohort.	Not stated but presumably R using the *lcmm* package.
8) Taylor et al. 2013 [44]	Bayesian SPJM.	*Model development and validation:* *N* = 3232, E = 458	Covariates include presenting PSA, T-stage, and Gleason grade. Longitudinal parameterisation includes PSA value & slope, and time-dependent HT.	Landmarks are given from most recent PSA values with a prediction window of three years.	External validation is performed on fourth dataset. Simpler visual approaches are undertaken, focusing on estimated risk of recurrence three years after treatment using a three-year prediction window. Patients are assigned to four risk groups, comparing the training and testing Kaplan-Meier plots, treating commencing hormone therapy as either censored and as an event. The model is deemed adequately calibrated with similar patterns being exhibited between training & testing datasets.	C
9) Proust-Lima et al., 2014 [45]	Frequentist JLCM & SPJM.	*Model development and validation:* *N* = 1178, E = 200	Biphasic mixed-effect parameterisation of longitudinal logged-PSA. Baseline covariates: presenting PSA, T-stage and Gleason. Four latent classes identified for the JLCM, SPJM included PSA value and slope association structure. All other components had the same model structure for direct comparison.	Landmarks taken at every six months from 1—3½ years, with a prediction window of three years.	Internal and external validation is performed. The EPOCE is estimated internally using CVPOL from 1 to 6 years after EBRT. The difference in EPOCE for 4-JLCM and SPJM shows the 4-JLCM to be a better prognostic model in the first four years. External EPOCEs and integrated BS are shown over the follow-up period. The IBSs and EPOCEs show reduced errors for ≥ 3-JLCM and SPJM with little difference between the two approaches.	R: using the *lcmm* and *JM* packages – code is available on request from authors.
10) Sène et al., 2016 [46]	Frequentist SPJM.	N = 2386, E = 312	Similar to [42] with biphasic longitudinal components for the logged-PSA, considering presenting PSA, T-stage, Gleason, and corrected total EBRT dose. Several specifications of the time-dependent initiation of salvage HT, and the association structures of the longitudinal value and slope of PSA and random effects.	Landmarks from 1.2, 1.6, 2 and 2.6 years are given with a prediction window of recurrence within the next three years. The predicted recurrence probabilities are given under four scenarios of initiating salvage HT immediately, in 1 or 2 years, or not at all.	Internal validation is performed using cross validation for a prediction window of three years. The CVPOL, CV-BS, and CV-IBS are shown for the six model structures. A simpler random effects joint model is best and chosen for the absence of salvage HT; for immediate HT, the JM that separated the PSA trajectory before and after HT is deemed best.	R: *JM* package with modifications to source code.
11) Ferrer et al., 2016 [47]	Frequentist multi-state SPJMs	*N* = 1474; E = 941^*^ * sum of all events	Similar to [42] with biphasic longitudinal components for the logged-PSA, considering presenting PSA, T-stage, and Gleason. The longitudinal PSA value and slope was modelled.	For the multi-state process, transition probabilities are given from each transition to any of the other four transitions from the end of treatment throughout follow-up.	A simulation study was undertaken to ensure the estimation process was correct. Diagnostic plots for the residuals and observed/predicted of the longitudinal model.	R: *nlme*, *survival*, *mstate* and *JM* packages, with adaptations. Code is readily available at the author’s GitHub account.
12) Ferrer et al., 2018 [48]	Frequentist landmarking and cause-specific SPJMs.	Not explicitly stated but as above. *N* = 1474; E = unknown.	Longitudinal logged-PSA modelled similarly as to [47]. Adjusting for: dataset cohort, age, T-stage, Gleason and presenting-PSA.	Predicted recurrence and competing risk of death probabilities for two patients at their landmarks of 1.3 to 2.5 years using a prediction window of 1½ and three years, comparing JM and landmark modelling.	Simulation study using the prostate patient cohorts to generate similar data. Evaluating robustness of JMs and landmark models, under different assumptions. JMs generally more robust to deviations in assumptions than landmark models, other than a strong violation in the longitudinal PSA biomarker specification where the landmark model performs better.	R: *nlme*, *JM*, *survival, pseudo*, and *geepack* packages. Code is readily available at the author’s GitHub account.

*Abbreviations*: *JLCM* Joint latent class model, *SPJM* Shared-parameter joint model, *PSA* Prostate-specific antigen, *EBRT* External-beam radiotherapy, *HT* Hormonal therapy, *EPOCE* Expected prognostic observed cross-entropy, *CVPOL* Cross-validated prognostic observed log-likelihood, *IBS* Integrated Brier score

## Methods

### Literature search strategy

**Fig. 1:**
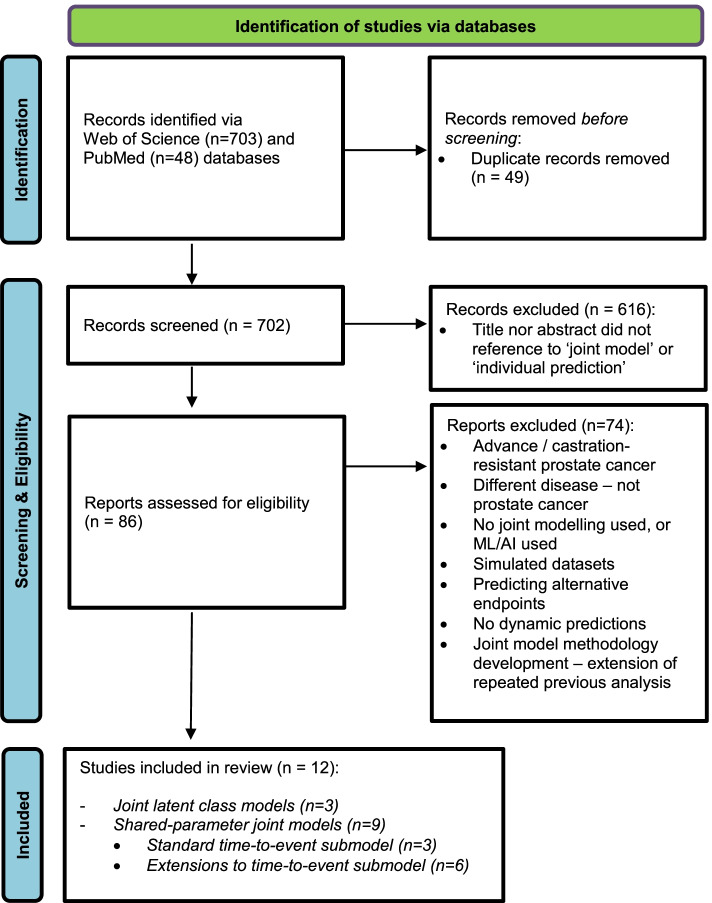
A flowchart for identifying studies of the literature review

Our search strategy included linear combinations of, {“joint model*” OR “individual* prediction”} AND {“prostate cancer” OR “prostate-specific antigen” OR “PSA”} in the title or abstract, using Web of Science and PubMed databases up to and including June 2020. A flowchart depicting the study identification strategy is given in Fig. 1. A total of 751 articles were identified from the initial search parameters, 703 and 48 articles came from Web of Science and PubMed respectively. Duplicated articles were removed leaving 702 unique papers. Novel and seminal papers that involve the joint modelling of the longitudinal biomarker PSA and time-to-event of clinical recurrence in localised prostate cancer were selected by the lead author, and selection discussed with co-authors, as the focus was to understand the PSA dynamics for this disease, which can be quite different from PSA dynamics for advanced prostate cancer. Further exclusions were made on inspecting the abstract, these included: advance/metastatic disease; different disease; no joint modelling undertaken, or alternative machine learning/artificial intelligence methods used; simulated data used; predicting alternative endpoints such as time to diagnosis or death; no dynamic predictions derived; and whether focus was on methodology development.

### Notation

In this section, we define the mathematical notation common to SPJMs & JLCMs frameworks. Let ***y***_*i*_ = y_i_(t_ij_); i = 1, …, N; j = 1, …, n_*i*_} be a longitudinal response vector of the continuous biomarker measurements for the *i*^*th*^ patient and *j*^*th*^ biomarker reading taken at time *t*_*ij*_. There are N patients with *n*_*i*_ longitudinal measurements per patient.

Let the random variable *T*_*i*_ be the time-to-failure for the *i* ^th^ patient, where ${T}_i=\min \left({T}_i^{\ast },{C}_i\right)$. The true event time is denoted ${T}_i^{\ast }$ and *C*_*i*_ is the censoring time. An indicator variable ${\delta}_i=I\left({T}_i^{\ast}\le {C}_i\right)$ is unity if the event of interest is observed for that patient, or zero otherwise.

### Shared-parameter joint model

Under the shared-parameter joint models framework, random effects are used to link the longitudinal and time-to-event components under study, whilst also accounting for the correlated repeated measurements within the longitudinal outcome.

The longitudinal process ***y***_*i*_ is assumed to follow a mixed-effects model, defined by a linear combination of possibly time-dependent main- and random effects *Y*_*i*_(*t*_*ij*_) = *m*_*i*_(*t*) ***+ ϵ***_*i*_(*t*_*ij*_) ***= β****X*_*i*_(*t*_*ij*_) + ***b***_*i*_*Z*_*i*_(*t*_*ij*_) + ***ϵ***_*i*_(*t*_*ij*_). The vector 𝜷 are coefficients for the main- and time-effect covariates of the design matrix *X*_*i*_, and the corresponding random effects ***b***_*i*_ for the *Z*_*i*_ design matrix. The measurement errors ${\boldsymbol{\epsilon}}_i\left({t}_{ij}\right)={\left\{{\epsilon}_i\left({t}_{i1}\right),\dots, {\epsilon}_i\left({t}_{i{n}_i}\right)\right\}}^T$ are independent and identically distributed and assumed to follow ${\boldsymbol{\epsilon}}_i\left({t}_{ij}\right)\sim N\left(0,{\sigma}_e^2\right)$, or t-distribution with several degrees-of-freedom, with the fatter tails used to accommodate for possible outliers. The random effects, independent of ***ϵ***_*i*_(*t*_*ij*_), are usually assumed to follow a multivariate normal distribution, with an unknown square covariance matrix structure *D*, ***b***_*i*_~MVN(**0**, *D*).

A relative risk, or proportional hazards model, is used for the parameterisation of the survival submodel:

$$\begin{aligned} {h}_i\left(t|{\boldsymbol{M}}_i(t),{\boldsymbol{w}}_i\right) &= \underset{\Delta t\to 0}{\lim}\frac{\Pr \left\{ t \le {T}_i^{\ast }<t+\Delta t\ \right|{T}_i^{\ast}\ge t,{\boldsymbol{M}}_i(t),{\boldsymbol{w}}_i \}} {\Delta t}\\ \\ {} &={h}_0(t)\exp \left\{{\boldsymbol{\gamma}}^T{\boldsymbol{w}}_i+f\left({\boldsymbol{M}}_i(t),{\boldsymbol{b}}_i,\boldsymbol{\alpha} \right)\right\}. \end{aligned}$$

Where ***M***_*i*_(*t*) denotes the true (unobserved) and entire longitudinal biomarker history up to time point t, with *m*_*i*_(*t*) indicating the true value at t (i.e. the mixed effect model not contaminated with measurement error). The baseline covariates in the hazard submodel are ***w***_*i*_, with ***γ***^*T*^ corresponding to the log-hazard ratio coefficients. An example of the parameterisation of the functional form *f*(***M***_*i*_(*t*), ***b***_*i*_, ***α***) can be a linear combination of value and gradient of the longitudinal biomarker, $f\left(\dots \right)={\alpha}_1{m}_i(t)+{\alpha}_2\frac{\mathrm{d}{m}_i(t)}{\mathrm{d}t}$. The corresponding ***α*** parameters quantify the intensity of association between the two outcomes. Other functional forms of *f* exist, such as the (weighted) cumulative effect (1), or random effects association (2),

1 $$f=\alpha\int_0^t\omega\left(t-s\right)\;\times\;m_i\left(s\right)\;ds$$

2 $$f=\boldsymbol\alpha^T{\boldsymbol b}_i$$

The former quantifies the risk of recurrence from the area under the biomarker trajectory and can allocate greater weights to more recent biomarker observations, e.g., using a standard normal density function for *ω*. The latter parameterisation uses only the random effects as a linear predictor, this requires no numerical integration which is computationally advantageous. Using a simple random intercept- and slopes structure is most interpretable, whereby patient deviations from the population average is expressed [49]. More elaborate structures are challenging to interpret [29, 50, 51]. A full parametric specification of the baseline hazard function, *h*_0_(*t*), is recommended (e.g. using constant-piecewise, or regression splines models), with an adequate number of knots for flexibly modelling the underlying baseline risk. Leaving *h*_0_(*t*) unspecified can lead to underestimating the precision of parameter estimates [52].

Given the random effects ***b***_*i*_, *Y*_*i*_ and *T*_*i*_ become independent (conditional independence). Excellent overviews of shared-parameter joint modelling can be found in Rizopoulos [49] and Papageorgiou et al. [29].

### Joint latent class model

The joint latent class models framework assumes the existence of latent classes that capture the association between the longitudinal biomarker trajectory and the relative risk of the endpoint of interest. Following the same notation as above, we can define the JLCM by the mixed-effect- and relative risk submodels for each latent class ***c***_*i*_ ∈ {1, .., *G*}^T^:

$${\displaystyle \begin{array}{c}\left({Y}_i\left({t}_{ij}\right)|{\boldsymbol{c}}_i=g\right)={\boldsymbol{\beta}}_g{X}_i^T\left({t}_{ij}\right)+{\boldsymbol{b}}_{ig}{Z}_i^T\left({t}_{ij}\right)+\epsilon \left({t}_{ij}\right); \phantom \epsilon \left({t}_{ij}\right)\sim N\left(0,{\sigma}_e^2\right),{\mathbf{b}}_{\mathrm{ig}} \sim \mathrm{MVN}\left({\boldsymbol{\mu}}_g,\mathrm{D}\right)\\ \\ {h}_i(t\ |{\boldsymbol{c}}_i=g)={h}_{0g}(t)\exp \left({\boldsymbol{\gamma}}_g^T{\boldsymbol{w}}_i \right)\end{array}}$$

where assignment to latent class *g* is given by a multinomial submodel,

$$\Pr \left({\boldsymbol{c}}_i=g\ |\ {X}_i\right)=\frac{\exp \left({\boldsymbol{\lambda}}_g^T{X}_i\right)}{\Sigma_{j=1}^G\exp \left({\boldsymbol{\lambda}}_j^T{X}_i\right)}.$$

With *X*_*i*_ a fixed baseline design matrix associated with classification and corresponding coefficients ${\boldsymbol{\lambda}}_g^T=\left({\lambda}_0^T=0,{\lambda}_1^T,\dots, {\lambda}_G^T=0\right)$. Given the latent class ***c***_*i*_**,** conditional independence between the longitudinal and time-to-event outcomes is assumed.

The JLCM has some advantages compared to the SPJM: it does not need to specify a suitable functional form to link the two processes, and thus the conditional independence assumption in the JLCMs results in less onerous computations. However, as the number of latent classes are not known a priori, it is another component to be estimated, and as these are not observed, the conditional independence assumption is nontrivial to evaluate. Jacqmin-Gadda et al. [43], proposed a trivariate score test to evaluate this assumption, they showed that their score test was uniformly most powerful and simpler than all other considered tests.

### Dynamic predictions

Given a sample from the population of interest, joint models permit to compute dynamic predictions of the event of interest at a future time *u* given the information available up to time *t* > 0. For a specific *i*^th^ individual, these are defined by *π*_*i*_(u| t) = Pr(*T*^∗^ ≥ *u* |*T*^∗^ > *t*, *X*_*i*_, ***y***_*i*_(*t*), *T*_*i*_, *δ*_*i*_, ***w***_*i*_, ***θ***). I.e., the conditional probability of being event-free at time *u* > *t*, given that the patient is still at risk of the event at time *t* (*T*^∗^ > *t*), the baseline covariates / fixed effects design matrix *X*_*i*_, the biomarker longitudinal values observed up to time *t*, ***y***_*i*_(*t*), and the parameters ***θ*** estimated from the joint model. These predictions, which can be then *dynamically* updated when new biomarker information becomes available at *t*^’^ > *t* [28].

For the shared-parameter JMs, these are extracted by integrating the conditional event probability *π*_*i*_(u| t) over the random effects. Similarly for the JLCM, the predicted probabilities are given by summing over the latent classes. In both frameworks, this is difficult to compute analytically, therefore Markov chain Monte Carlo (MCMC) methods are implemented. MCMC extracts the predicted event posterior distribution of *π*_*i*_(u| t) and corresponding credible intervals from the Monte Carlo sample percentiles of interest [45, 53].

### Predictive performance

Measuring predictive ability is crucial to assess the proposed model(s) performance in producing accurate predictions, the end goal for any DPM (dynamic prediction model). Two aspects of modelling performance can be assessed: calibration (how well the model predicts the observed data) and discrimination (how well can the model distinguish between those patients that do and do not have an event).

Discrimination is typically assessed by considering the time-dependent AUROC (area under the receiver operating characteristic curve) [53–56]. Within a particular chosen prediction window, AUROC (or simply AUC) values of 0.5 indicate random chance assignment and values closer to unity indicate better model discrimination.

The prediction error (PE) focuses on assessing the calibration of the model, and it is defined as the expectation of the difference between the observed event status ${N}_i\left(u|t\right)=I\left({T}_i^{\ast }>u|t\right)$ and the predicted event occurrence *π*_*i*_(*u*| *t*), at a specific time. A loss function can be incorporated within the expectation, e.g., the absolute- or mean squared-loss functions. The latter is also known as the Brier score (BS), which is an overall measure of prognostic performance [57, 58]. Under any loss function, as the difference between these two terms decrease and tends to zero, the closer the observed and predicted event align, resulting in better predictive performance of the model. In practice, one may want to consider predictions over a window of interest, rather than specified time points, by using weighted extensions of these estimators, e.g. weighted average absolute prediction error (WAPE) or integrated BS [59, 60]. For any of these predictive measures to be valid, the censoring distributions need to be corrected for, e.g. using inverse probability weighted estimators [28, 56, 61, 62].

Alternative measures of accuracy can be utilised, such as the expected prognostic observed cross-entropy (EPOCE) [63]. The EPOCE quantifies the prognostic information from the joint model at the landmark time of interest. When estimated internally, leave-one-out cross-validation of the prognostic observed log-likelihood (CVPOL) is used to correct for over-optimism [64]. For external validation, no cross-validation is required. Proust-Lima et al. [45] argue the advantages of EPOCE over the previously stated measures, including no censoring distribution nor a prediction window is assumed, direct comparison of two joint models can be made, and that it is more reasonable to evaluate directly on the likelihood density functions. Further formulation and discussion on this predictive accuracy metric can be found in [45, 63].

## Results

We identified 12 relevant full-text papers that best illustrated the joint modelling framework and summarised its applications in localised prostate cancer, these were selected to be included within this review. Table 2 summarises these twelve papers including details of the modelling framework used, sample sizes, parameterisations, the prediction windows of interest, whether validation was undertaken, and the code/software used.

Where available, the corresponding software and code with packages can also be found [65, 66]. Nine papers (9 of 12, 75%) applied the shared-parameter joint modelling framework, with three of these presenting the standard joint model for a time-to-failure endpoint, while 6 of 9 papers presented extensions to the time-to-event submodel incorporating cure, competing risks, and multi-state models for localised prostate cancer (e.g., local- and distant recurrence, salvage therapy, and death). Three papers (3 of 12, 25%) described the joint latent class approach. In the following, we review and summarise these papers in detail around their model specification, estimation of dynamic predictions and model validations conducted.

### Shared-parameter joint models to predict recurrence in localised prostate cancer

In this section, we focus our review on three relevant papers that investigated PSA dynamics to predict recurrence in localised prostate cancer using the SPJM framework: Taylor et al. [44], Sène et al. [46], and Pauler & Finkelstein [37]. All three articles develop models in localised prostate cancer patients treated with EBRT in the absence of neoadjuvant HT. Taylor focused on developing a model to creating a clinical prediction tool online; Sène explored the effect on initiating salvage treatments at different time points and its effect on the predicted dynamic probabilities of recurrence. Pauler & Finkelstein use a change-point model to capture any jump in PSA.

#### Model specification

In Taylor et al. [44], the functional form over time of the longitudinal PSA mixed model assumes three phases: baseline/presenting PSA (Β_0_), and the short-term (decrease, Β_1_)**,** and long-term (increase, Β_2_) evolutions of PSA, *Y*_*i*_(*t*) = log[PSA_i_(*t*) + 0.1] = Β_0_ + Β_1_*f*_1_ + Β_2_*f*_2_, with ${f}_1=\left\{{\left(1+\mathrm{time}\right)}^{-\frac{3}{2}}-1\right\}\ \mathrm{and}\ {f}_2=\mathrm{time}$. For each of the three phases, *B*_*k* = {0, 1, 2},_ are matrices containing linear combinations of the fixed baseline covariates T-stage, Gleason grade and presenting pre-treatment PSA, along with subject-specific random effects parameters. A t-distribution with five degrees-of-freedom for the error term is assumed. Time to prostate cancer clinical recurrence is measured from the end of RT. In the survival submodel, the functional form *f*(***M***_*i*_(*t*), ***b***_*i*_, ***α***) is a linear combination of the value of PSA concentration and its slope at time t, $f\left({\boldsymbol{M}}_i(t)\ {\boldsymbol{b}}_i,\boldsymbol{\alpha} \right)={\alpha}_1\mathrm{PSA}(t)+{\alpha}_2\frac{d\ \mathrm{PSA}(t)}{dt}$. Additionally, the survival submodel included a time-dependent indicator variable for when salvage hormonal treatment (ST) is initiated to account for the subsequent drop in hazard of clinical failure. PSA values after ST were excluded due to the sudden decrease in PSA trajectory and did not feature in the mixed-effect model; however, clinical recurrences after ST were considered. A piecewise constant function is assumed for the baseline hazard.

Sène et al. [46] made similar modelling assumptions as Taylor et al. [44] for the functional forms in the mixed and survival submodels. The model adjusted for presenting PSA, Gleason score, T-stage, and total corrected dose of EBRT (using the linear-quadratic model given in [67]). Initiation of ST was included as a time-dependent indicator variable to reflect the potential decrease in risk of progression; five functional forms of ST were considered. Three different association structures of *f* were fitted: a linear combination of PSA value and gradient (with and without a logistic transformation for PSA), and the random effect structure, which evaluated the individual deviations from the overall population’s PSA trajectories. A combination of those different parametrisations yielded 12 models with varied complexity.

Pauler & Finkelstein [37] proposed a change-point parameterisations in the longitudinal model for PSA, by incorporating an unknown change-point indicator variable for whether change in PSA has occurred. If a shift is indicated, a likely change-point time-range is estimated with a uniform distribution for PSA. A narrower posterior change-point range with larger differences in the slopes before- and after the change-point indicate prostate cancer recurrence is likely (before the formal clinical failure endpoint). Trivariate normal and uniform priors are used for four random effects, they included: intercept; change-point time (uniform); the slope before and after the change-point. For the survival submodel, a piecewise exponential hazard function was used. Baseline covariates included age, presenting PSA, and disease stage. For the joint model, non-informative priors were chosen.

#### Estimation, prediction and validation

In Taylor et al. [44], estimation was undertaken under a Bayesian framework using C software. The joint model was developed on three pooled cohorts (totalling *N* = 2,386 patients) and externally tested using a separate fourth dataset (*N* = 846 patients). Dynamic predictions for an individual patient’s PSA trajectory and risk of recurrence for the next 3 years were shown: no formal validation measures were presented. The authors opted for simpler graphical inspections to study the model, owing to the complicated nature of the time-dependent ST events within the validation cohort. An online prognostic calculator was developed, enabling individual dynamic predictions of disease recurrence given PSA trajectories for future patients (http://psacalc.sph.umich.edu^1^).

In Sène et al. [46], estimation was undertaken under a frequentist framework, and R software used for model development, again using the same three cohorts as in Taylor et al. [44]. Internal approximated leave-one-out cross-validation was used to assess six of the 12 models’ predictability, using BS and EPOCE accuracy measures [64]. The two best fitting models were the logistic-transformed PSA value and slope that separated the effect of PSA before- and after ST, whilst the model with the random effect association structure performed best when assumed that the patient would not start ST within 3 years. Exemplar individualised dynamic predictions used a prediction window of 3 years on an intermediate risk patient. Different scenarios when ST would be initiated were used to illustrate the impact of delays in ST initiation on risk of recurrence. External validation was not performed.

We cannot make direct comparisons between the predictive performances of the two papers as they used different assessment methods (graphical approaches in Taylor, EPOCE & BS presented in Sène). In Sène et al., patients who did not receive HT nor ST within 3 years were mainly used in order to assess predictive performance. Sène noted that this may not be a representative situation for all patients, so they performed a sensitivity analysis using Taylor’s approach to widen the sample on HT-free patients at the landmark prediction time only, then with subsequent ST initiation within the three-year prediction window, as either a recurrence event or dependent censoring. The relative predictive performance was largely unchanged in both papers under this approach and therefore can be considered robust.

In Pauler & Finkelstein [37], estimation was done in a Bayesian framework, using C and S-plus software. The joint model was developed on a cohort of *N* = 676 patients. As the majority of patients do not exhibit clinical failure, the slope after the change-point was non-significantly negative, indicating PSA trajectories generally remain constant over the follow-up period. The regression coefficients from the relative risk component are not straightforward to interpret due to the number of pairwise and three-way interactions, the authors noted that coefficients are in the expected directions. Sensitivity analysis was done on three differing definitions of recurrence based on PSA rises. They showed that regardless of rule followed, there was little difference to their optimal joint model. The AIC rose when considering only a relative risk model using indicator covariates for each rule, this provided justification on using the joint change-point model, as the longitudinal PSAs substantively improve the goodness-of-fit. The posterior distributions of four individual patient change-points were shown. For two patients who do relapse, sharp change-points are given between 2 and 4 years, who then go on to recur at six and 4 years of follow-up. For stable PSA patients, the change-point is imprecise with very wide uniform posteriors. Individualised predictions are performed on two hypothetical patients showing each’s posterior predictive distributions of time to relapse. Although discussed, the model was not validated.

### Latent class joint models to predict recurrence in localised prostate cancer

In this section, we focus our review on relevant papers that investigated PSA dynamics using the JLCM framework. There are three papers of interest reviewed in this section, by Proust-Lima & Taylor [42], Jacqmin-Gadda et al. [43], and a third paper by Proust-Lima et al. [45], which is appraised separately as this compares the SPJM and JLCM.

Proust-Lima & Taylor [42] modelled the functional longitudinal PSA similarly to Taylor et al. [44] (described previously). Baseline covariates T-stage, Gleason score, and pre-treatment PSA were included into both submodels. The survival submodel also includes an exogenous time-dependent indicator variable for initiation of ST, and a class-specific Weibull baseline hazard function.

Model development was performed on a single cohort of patients (*N* = 1,268), and external validation was performed on two additional smaller cohorts (with *N* = 503 and *N* = 615 patients respectively). Several JLCMs were fitted with ranging classes (2—6), with the five-class model (5-JLCM) producing the lowest Bayesian Information Criterion (BIC); the optimal model included estimation of 75 parameters. Predicted PSA evolutions and survival curves for each of the five classes illustrate how PSA trajectories with long-term rise of PSA correspond to greater risk of failure. Dynamic predictions were made within a prediction window of 3 years for two patients with contrasting baseline risk factors: a lower-risk patient who recurs and a higher-risk patient with no observed recurrence.

Within each external validation cohort, measures of predictive accuracy (absolute prediction errors EP and WAEP) for the five-class JLCM were computed, and compared to a relative risk model with baseline information only, and a two-stage landmark model. The JLCM was shown to be the best fitting at various landmark times, and accounting for the longitudinal biomarker reduced both the EP and WAEP, particularly at earlier landmarks.

For Jacqmin-Gadda et al. [43], the score test methodology introduced previously is applied to develop a prognostic joint model for prostate cancer recurrence (with the same dataset used as in Taylor et al., [40]). They develop the JLCM similarly to Proust-Lima et al., [35, 42]. They show that the more flexible 4-class JLCM did not reject conditional independence, whereas the less powerful alternative Wald test for dependence failed to reject the null for a 3-class JLCM.

#### Comparison between latent-class and shared-parameter joint models

A direct comparison is made between the two types of joint models applied to prostate cancer by Proust-Lima and colleagues [45]. Three prognostic baseline factors were adjusted for, logged initial-PSA, T-stage, and Gleason score using the same Michigan hospital cohort dataset. The three-component parameterisation of PSA in the mixed-effect model was done in the same manner to Proust-Lima & Taylor, and Taylor et al. [42, 44] for both joint models for direct comparison. The developed 4-JLCM adjusting for PSA value and slope was chosen from information criteria and conditional independence being met. The BIC favoured the 4-JLCM compared to the shared-parameter JM.

For direct comparisons between the JLCM and SPJM, evaluation of dynamic predictions (for the entire follow-up) are made using the cross-validated EPOCE framework in the first 6 years. The 4-JLCM is superior to the SPJM in the first 4 years on internal validation, and also slightly better in the first 3 years on external validation.

### Extensions to the shared-parameter joint model

We present some further extensions to the joint model in the following subsections. In particular we comment and review four papers with a cured fraction [38–41]; a competing risk joint model [48], where clinical recurrence is competing with a non-related cancer death; and a multi-state joint model [47], whereby patients can go through a pathway of disease states throughout follow-up.

#### Joint-cure models

A natural extension to the SPJM is to incorporate a cure component to the time-to-event submodel, whereby patients are considered to be susceptible to experience the event under study (e.g. recurrence), or, on the contrary, to be cured after initial treatment, and thus never susceptible of recurrence. Allocation into the two groups is typically modelled using a logistic classifier submodel:

$$\Pr \left(D=1|\ {X}_i\right)=\frac{\exp \left({\boldsymbol{\beta}}^T{X}_i\right)}{1+\exp \left({\boldsymbol{\beta}}^T{X}_i\right)},$$

where *D* = 1 refers to the susceptible group (observed only when the event of interest occurs), *X*_*i*_ is the fixed baseline design matrix with their corresponding vector of coefficients, ***β***. Patients that have been allocated to the ‘cured’ group are coded *D* = 0.

There can be a high proportion of patients that are recurrence-free after long follow-up, resulting in heavy censoring. This may compromise the predictions of a joint model given the lack of events observed. It therefore may be appropriate to model these patients that appear to have prolonged event-free survival as ‘cured’, using a cure joint model.

There are four articles that consider a joint cure model for the risk of clinical recurrence [38–41]. The four papers have a similar model specification: a nonlinear parametric exponential decay-growth (U-shaped) model is used to capture the logPSA trajectory *m*_*i*_(t, **r**_*i*_) = *r*_*i*1_ exp(−*tr*_*i*2_) + *r*_*i*3_ exp(*tr*_*i*4_), where *r*_*i*1, …, 4_ are the random effects to be estimated. Those that have been allocated to the cure group (from the logistic incidence submodel) have *r*_*i*4_ ∣ (*D* = 0) = 0, as this assumption reduces the PSA trajectory, *m*_*i*_(*t*, ***r***_*i*_), to an exponential decay cure SPJM. The conditional failure time model is given by *h*(*t*| *D*_*i*_, *X*_*i*_, ***r***_*i*_, ***β***, ***α***, *g*(*m*_*i*_)) = *h*_0_(*t*) exp( ***β***^*T*^*X*_*i*_ + ***α****g*(*m*_*i* ∣ *D*_, *t*)), where *g*(*m*_*i*_) can be given by including the trajectory function and its slope given in [40, 41].

Baseline covariates included pre-treatment PSA, T-stage, and Gleason score. Additionally Taylor et al. [40] considered PSA value & slope as time-dependent covariates, age, EBRT total delivered dose (in Gy) and treatment duration as baseline covariates. Yu and colleagues [41] included an exogenous time-dependent variable to indicate start of salvage HT, similarly to [44], and used a generalised Weibull model for the baseline hazard function. Both frequentist and Bayesian approaches are directly compared by Yu et al. [39].

In Law et al. [38], the joint cure model is compared to the standard cure model without longitudinal time-dependent information, and to the shared parameter joint model without the cure component. They showed better predictions and discrimination, together with reducing biases from informative censoring. Taylor et al. and Yu et al. [40, 41] compared the predictions of the model with updated information on the same patients who were initially used to develop the model, that is whereby more longitudinal PSAs and events on the same patients are gathered.

The extended shared-parameter joint-cure model offers additional flexibility to model the inherent heterogeneity of patients that go on to have extended event-free survival. Yu et al. directly compared joint models with and without a cure component. They showed a standard JM tends to overestimate the number of clinical events. They compared the two models using the conditional predictive ordinate and BIC, both favouring the additional cure submodel component, despite an extra 30 parameters needed to be estimated [41]. This however may over-parameterise the model without adequate event sizes [68]. Also as the prostate cancer disease pathway is complicated, clinical input is recommended with regards to plausibility of the cure component and its definition.

#### Competing risks joint models

The event of interest may be precluded by the occurrence of a competing event, for instance, non-cancer related deaths before recurrence. It is well known that biases are elicited by censoring these competing event deaths [69, 70]; joint models can be extended considering the presence of a competing event.

Ferrer et al. [48] perform individual dynamic predictions and validate the robustness of the estimators in the presence of competing risk of death (from a non-related cancer cause), within a frequentist framework. A cause-specific proportional hazards submodel is proposed for each competing event, and thus the relationship of the longitudinal biomarker with each competing event can be assessed. Individual dynamic predictions were estimated and compared to landmarking estimators. Two simulation studies were performed using simulated data that was alike to the applied prostate cancer dataset. Each approach validated the estimators, then compared and assess their robustness to misspecification of the joint model. Both the AUC and mean-squared prediction error were employed to characterise the predictive accuracy. An extension of the AUC was adapted to the competing risk setting, proposed by Blanche et al. [61]. It was shown that in almost all cases, the joint models were superior to the landmark models. The landmark models were only superior to the joint models when the longitudinal biomarker was heavily misspecified. Ferrer’s competing risk paper is the only study to present validation metrics, using simulated studies. Code is available at https://github.com/LoicFerrer/Individual-dynamic-predictions.^2^

#### Multi-state joint models

The evolution of localised prostate cancer over time can be characterised by the occurrence of different events of interest, such as biochemical failure, local recurrence, distant recurrence and death. One way to jointly model all these events is via multi-state models, in which the event progressions of interest define the transition between different disease states [71]. As longitudinal process such as PSA trajectories can have an impact on several of these event transitions, multi-state models can be generalised to the joint modelling framework.

Ferrer et al. [47] proposed modelling the longitudinal PSA process using a mixed-effect submodel, similarly to Proust-Lima and Taylor [42], Sène et al. [46], and Ferrer at al [48]. They used a non-homogeneous Markov multi-state model for the intensity of the transitions between five states: 0) end of EBRT treatment, 1) local recurrence, 2) salvage HT, 3) distant recurrence, and 4) death (the absorbing state). Intermediate states could be skipped (e.g. ending EBRT_0_ ➔ death_4_), and backward transitions were not allowed. Two properties were considered: 1) the Markov property whereby the future process is only dependent on the present state and not the preceding transitions / states; 2) the non-homogeneous property ensures the time since entering the study influences the evolution of the process.

Each transition intensity is modelled assuming proportional hazards and incorporated the biomarker trajectory. For each transition from state *i* to *j*, only patients visiting the state *i* are included in the analysis. The baseline intensity function was modelled parametrically. The maximum likelihood framework was used to estimate the corresponding parameters.

The multi-state joint model was fitted with the same two study datasets as in Ferrer et al. [48]. Four covariates (presenting PSA, Gleason score, T-stage, and study cohort) were adjusted for in the models. Worse baseline risk factors were associated with higher values in their long-term PSA trajectories, and reaching clinical failure states earlier. Higher presenting PSA was associated with a higher instantaneous risk to salvage hormone therapy or death. A linear combination of increases in PSA value & slope were associated with significant deterioration for all clinical progression transitions (local, salvage HT, or distant failures) from the initial state.

After adjusting for all other covariates and PSA slope, a unit increase in the log PSA gave rise to a 43% increase in risk to local recurrence. Patients with continually high PSAs or increasingly steep PSA gradients after EBRT treatment, led to earlier and higher hazards to clinical failure states. Conversely, higher PSA levels had a protective effect on the transition to direct death after EBRT but were more likely to progress though the prostate cancer progression states.

Predictions were compared with the observed data. The observed values were averaged at each decile with corresponding predicted values computed, they show the observed values lay within the 95% CIs, with very similar predicted values. The predicted transition probabilities over time, in a given state to another other feasible state are presented, comparing similar parametric estimated probabilities to the observed. The only exception was between transitions 1➔2 (from local recurrence to receiving HT) where the spike after EBRT was not adequately captured with the splines, it shows there is a very near-immediate initiation of HT after localised recurrence to control the disease. It is worth noting that PSA dynamics were only collected until the patient’s first clinical event and not thereafter and were extrapolated according to their posterior trajectories.

Diagnostics of the joint multi-state model were evaluated visually. Residuals vs fitted values, observed and predicted PSA trajectories, and predicted vs non-parametric transition probabilities between states were presented. In general, they showed the model fits particularly well to the longitudinal, and multi-state submodels. The models themselves were not externally validated nor stated any predictive performance measures, only the estimation process via simulation studies. Although equations for obtaining individual dynamic predictions for patients were presented in the paper, these were not demonstrated with specific examples.

The code to apply these multistate models to a simulated dataset and adapt for use is freely available at https://github.com/LoicFerrer/JMstateModel^3^ and could be used to derive patient predictions and be adapted for the reader’s need.

## Discussion

Over the last two decades there has been a plethora of research on PSA protein concentration and its association to recurrence, or prolonged event-free survival (effectively cure). We have reviewed and assessed 12 papers that report joint models of longitudinal PSA trajectories and time-to-event endpoints that aim to describe how these trajectories impact and predict prostate cancer recurrence. We found two broad frameworks (SPJMs & JLCMs) that were utilised and assessed different methodologies. We synthesize these different approaches applied to similar dataset cohorts of prostate cancer patients initially receiving EBRT without HT, which allow the methodology to be compared. Due to the long-term nature of prostate cancer recurrence and progression, the datasets to develop the DPMs comprise patients recruited from the 1980s. As long-term follow-up is necessary the historical nature of the datasets is unavoidable but the impact of changes in clinical practice should be considered when utilising DPMs for contemporary patients.

There are limitations to our work, as this report was not initially intended to be a systematic review on all the available literature, but a synthesised summary of what we considered relevant articles of modelling both PSA longitudinally, and time-to-recurrence in localised prostate cancer; in preparation for an application for these methods in our own dataset (in a publication to follow). For instance, we focused on specific key words within the title and abstract only, so we may have missed reports if the use of these terms was not explicit in these fields. Further joint modelling papers not included here were due to, for instance, no dynamic predictions presented [72], a mix of non-radiotherapy treatments (e.g, radical prostatectomy); methodology development focused but repeated analysis referred to [63, 73, 74]; or exclusive use of simulated datasets [75]. It was noted that not all papers were expectedly populated by the search strategy [76]. In the localised prostate cancer setting, where PSA is used to monitor recurrence after radical treatment of disease, joint models have also been used in the context of prostate cancer screening [77–82] or advanced (metastatic) disease [33, 83–87]. We did not consider these scenarios as the PSA dynamics differ greatly. These models could also be extended to accommodate more than one longitudinal biomarker, such as PSA and testosterone, or the sequential findings on digital rectal exams, in a joint multivariate model. Regardless of disease stage, these papers highlight and emphasise the use of longitudinal information, such as the PSA biomarker. This increases the prognostic power of the prediction model to help inform and update predictions of the event of interest, compared to solely using baseline risk factors that are imprecise [88].

Modern typical first-line treatment of localised prostate cancer include HT before (neoadjuvant) and concurrently with external-beam radiotherapy [12, 89], and PSA trajectories are known to be more homogeneous with combined treatment [44]. Furthermore, given recent advances in radiotherapy techniques and the use of moderate- and ultra-hypofractionation (fewer but larger doses of radiotherapy) [90, 91], treatment exposures of RT are where there are far fewer treatment exposures of RT than the average treatment durations presented in these papers. The tool in Taylor et al. [44] was developed in the absence of neoadjuvant HT, therefore predictions from these models have limited applicability within current treatment pathways. Further development of these models for patients receiving HT are needed.

The papers reviewed provide a very good exposition and rationale to their model development and clinical usage. Regardless of the functional form used in the joint modelling framework, a fully parametric form was fitted for the mixed-effects model. There are possibly more appropriate and flexible forms that may exist, compared to the biphasic form for PSA trajectories they postulate throughout [35, 42–46, 48]. Many of the reviewed articles present an appraisal of their models, either by validation or contain a simulation study. External validation is seen as the gold-standard, to ensure model suitability and generalisability in other patient populations and to assess overfitting [92]. However, when rigorous measures of predictive performance have not been reported in these papers, these would not be considered validated by today’s standards [93].

As with any specification of modelling, there are pros and cons to the joint modelling approach taken and several differences exist. For JLCMs, the maximum likelihood approach contains closed-form solutions and are computationally feasible to compute. They are advantageous for the use of developing a predictive joint model for dynamic predictions, whilst not having to impose specific parametric assumptions for the biomarker’s functional form (e.g. current value, slope, area), unlike SPJMs [45]. Robustness to deviations of the imposed functional form have been rigorously assessed in Ferrer at al [48]. In this paper, they demonstrated that no method (joint modelling nor landmarking) was particularly robust to misspecification in the longitudinal biomarker. However, when there was heavy misspecification, landmarking methods did perform better than joint modelling.

The SPJMs assume a homogenous population with a singular average PSA biomarker trajectory, whereas JLCMs account for further population heterogeneity through the latent classes. Both JLCMs and SPJMs account for the variability of the PSA biomarker through the random effects in the longitudinal model. The purpose of the random effects in the SPJM is two-fold, accounting for the correlation of the repeated measures in the mixed-effect model, and the association between the PSA biomarker and time-to-recurrence, whilst in the JLCM only the latent classes account of the association between the biomarker and event.

Disadvantages of the JLCMs approach include the possibility of having multiple local maxima for the maximum likelihood estimates, and several models are needed to be fitted in order to find the optimal number of latent classes (by comparing multiple information criteria) [49]. Some of these issues can be circumnavigated via parallelisation of the computation for more optimal resourcing, e.g., make use of parallel computing by using search grid methods for JLCMs as computations are independent (see the *mpjlcmm* function from R package lcmm); or implementing multiple MCMC chains performed in parallel using Bayesian SPJMs (*jm* function from R package JMbayes2) [65, 94].

Both Frequentist and Bayesian paradigms were used for the SPJMs, whereas we only reviewed frequentist methods for the JLCMs. In their direct comparison of JLCMs and SPJMs [45], the authors showed that the JLCMs had less assumptions and performed better. However when adjusting for the same patient cohort dataset, baseline covariates, prediction times, and biphasic components for the longitudinal PSA component: the prognostic accuracy measures for EPOCE in Sène et al. [46] using SPJMs appeared superior than those obtained with the JLCM in Proust-Lima et al. [45].

All models reviewed in this paper can produce dynamic predictions for prostate cancer prognosis. The JLCMs do not assume a specific association structure nor quantify those associations, (like the SPJMs do), they describe the trajectories in a heterogeneous population. If the main goal is to quantify the associations assuming a homogenous population, then SPJMs are recommended. There is not one overarching or standout model to always use by default. The choice of model may be primarily driven by the research question and personal choice. If the purpose is solely for prediction, then combining several frameworks for dynamic predictions using some weighted model averaging methodology could be applied [95]. Indeed one type of framework may outperform another at certain time intervals and then vice-versa at different time windows. Each model has its own advantages, depending on the end goal of the reader. It is hard to compare each model’s framework with another in terms of superior predictive performance as not all these papers present these metrics.

This review focused on radiotherapy, however there are other treatments for prostate cancer including hormone therapy, prostatectomy and combinations therein, though optimal timing of these combinational therapies appears unclear [96, 97]. There have been recent advances in using sophisticated machine learning/artificial intelligence (ML/AI) techniques on imaging data to predict whether patients require biopsies, or to predict clinical failure or death under these alternative treatment pathways. Some recent articles include development of artificial neural networks, support vector machines, and random forests for predicting diagnoses [98, 99], optimal timing of biopsies [100], and clinical failure [101] or death [102]. However, it is not apparent that the longitudinal nature of time-varying markers like PSA have been considered, nor produce dynamic predictions. A review of these AI and ML methods is given in Tătaru et al. [103]. Some authors refer to joint modelling itself is an AI approach [104]. Other studies have suggested combining the boosting approaches of machine learning to joint models, to create a unified framework using mechanistic data-driven approaches [105]. ML/AI techniques are not a panacea and need to be correctly developed and incorporate all available information, be rigorously validated, and to have clinical utility [106–109]. Reporting guidance, based on TRIPOD & PROBAST statements, have been developed for AI & ML (TRIPOD-AI/ML & PROBAST-AI/ML) [110–113].

## Conclusions

To conclude, we reviewed, summarised, and synthesised principal methodologies on twelve seminal papers over the last two decades on dynamic prediction joint models applied to the prognosis of prostate cancer patients, using PSA to dynamically update prognosis. This article supports the use of utilising longitudinally collected PSA, in addition to baseline prognostic factors to improve predictions in a joint modelling framework. There have been many advancements in computational processing, methodologies, with improvements in clinical practice and treatments. Combining all these developments together with utilising all available information, the future of dynamic prediction models is encouraging.

## Data Availability

Data sharing is not applicable to this article as no datasets were generated or analysed during the current study.

## Abbreviations

AI: Artificial intelligence
AIC: Akaike information criterion
AUROC (or AUC): Area under the receiver operating characteristic curve
BcF: Biochemical failure
BIC: Bayesian information criterion
(I)BS: (Integrated) Brier score
CI: Confidence interval (frequentist) *or* credible interval (Bayesian)
(CV)POL: (Cross-validated) prognostic observed log-likelihood
CPM: Clinical prediction model
DPM: Dynamic prediction model
EBRT: External beam radiotherapy
EP: Prediction error (or error of prediction)
EPOCE: Expected prognostic observed cross-entropy
HT: Hormonal therapy
JLCM: Joint latent class model
JM: Joint model
ML: Machine Learning
NCCN: National Comprehensive Cancer Network
PSA: Prostate-specific antigen
RT: Radiotherapy
SPJM: Shared-parameter joint model
ST: Salvage therapy
WAEP: Weighted average absolute error of prediction

## Footnotes

1 Last accessed in September 2022.

2 Last accessed in September 2022.

3 Last accessed in September 2022.
